# Complementary and alternative medicine use among older adults with musculoskeletal pain: findings from the European Social Survey (2014) special module on the social determinants of health

**DOI:** 10.1177/20494637211023293

**Published:** 2021-06-13

**Authors:** Ann-Marie Morrissey, Aoife O’Neill, Kieran O’Sullivan, Katie Robinson

**Affiliations:** School of Allied Health, Health Research Institute, Ageing Research Centre, University of Limerick, Limerick, Ireland

**Keywords:** Pain, musculoskeletal, older adults, complementary and alternative medicine, alternative therapies

## Abstract

**Background:**

This study describes the use of complementary and alternative medicine (CAM) among older adults who report being hampered in daily activities due to musculoskeletal pain. The characteristics of older adults with debilitating musculoskeletal pain who report CAM use is also examined.

**Methods:**

Cross-sectional European Social Survey Round 7 data from 21 countries were examined for participants aged 55 years and older, who reported musculoskeletal pain that hampered daily activities in the past 12 months.

**Results:**

Of the 4950 older adult participants reporting musculoskeletal pain that hampered daily activities, the majority (63.5%) were from the West of Europe, reported secondary education or less (78.2%), and reported at least one other health-related problem (74.6%). In total, 1657 (33.5%) reported using at least one CAM treatment in the previous year. Manual body-based therapies (MBBTs) were most used, including massage therapy (17.9%) and osteopathy (7.0%). Alternative medicinal systems (AMSs) were also popular with 6.5% using homoeopathy and 5.3% reporting herbal treatments. A general trend of higher CAM use in younger participants was noted. CAM use was associated with physiotherapy use, female gender, higher levels of education, being in employment and living in West Europe. Those reporting multiple health problems were more likely to use all CAM treatments, except MBBT.

**Conclusion:**

A third of older Europeans with musculoskeletal pain report CAM use in the previous 12 months. Certain subgroups with higher rates of CAM use could be identified. Clinicians should comprehensively and routinely assess CAM use among older adults with musculoskeletal pain.

## Background

High rates of musculoskeletal pain are reported by older adults,^1^ with its prevalence set to increase due to a globally ageing population. Musculoskeletal pain is associated with a number of clinical, societal and psychological consequences including lower activity levels,^2^ incident disability,^3^ increased occurrence of falls,^4^ depression and anxiety symptoms,^5^ frailty,^6^ reduced quality of life^7^ and increased healthcare utilisation.^8^

Despite its prevalence and associated burden, the management of musculoskeletal pain continues to be a major healthcare challenge.^9^ Traditional pharmacological approaches, such as the use of opioid analgesics, are no longer recommended, particularly in the long-term, due to the heightened risk of both adverse effects and treatment discontinuation.^10,11^ The implementation and use of management strategies, in particular opioid alternatives, for musculoskeletal pain should be evaluated to understand their use, quality and effectiveness. This includes CAM, a commonly reported management strategy for pain among older adults.^12,13^

CAM covers a diverse group of therapies, not considered to be a part of mainstream medical care and which are typically seen as being health-related.^14^ While there is no globally accepted definition for CAM, operational definitions typically involve identifying specific modalities and grouping them into domains.^15,16^ The National Institutes of Health (NIH) National Centre for Complementary and Integrative Health (NCCIH)^15^ categorise CAM into one of these three domains: (a) natural products; (b) mind and body practices; (c) other complementary health approaches. In 2011, the Cochrane Collaboration developed an official list of modalities to be considered within the CAM field’s scope and categorised them in the following domains: Mind-Body medicine, Natural Product-based Therapies, Energy Medicine, and Whole Medical Systems.^16^

CAM usage has increased as a result of growing dissatisfaction with traditional medicine;^17^ however, its legislation, regulation and accreditation continue to vary considerably between regions.^18,19^ CAM usage is common among the general population across Europe^20^ and is associated with health, socioeconomic and demographic indicators.^21^ A review of surveys of CAM use in the United Kingdom found an average 1-year prevalence of CAM use of 41.1% and an average lifetime prevalence of 51.8%.^22^

Systematic reviews have concluded there is no compelling evidence for the effectiveness of various CAM interventions to comprehensively manage musculoskeletal pain beyond the short term, including osteopathy,^23^ chiropractic interventions,^24^ herbal medicine,^25^ acupuncture^26^ and massage therapy.^27^ Concerns have also been raised about the risks of CAM and adverse outcomes, including the risk of allergic and anaphylactic reactions,^28^ CAM-induced acute liver failure and injury,^29^ intoxications^30^ and serious complications of chiropractic manipulations.^31^ Though evidence is lacking a review of CAM use for back pain found prevalence rates of consultations with one or more CAM practitioners ranging from 37% to 76.4% (mean: 55.2%; median 53.6%) across five studies drawing on nationally representative samples.^32^

Variations in rates of disclosure of CAM by patients to healthcare providers have been identified with reasons for non-disclosure including lack of inquiry by healthcare providers, belief that providers would support CAM use, belief that disclosure was important for safety and belief providers would advise about CAM use.^33^ A study of older adults’ reasons for CAM nondisclosure to physicians found older people were less likely to report ingestible types of CAM use compared to physical or mind/body types of CAM.^34^

Given the high rates of CAM use among older people, the unique clinical challenges of managing pain in this population, the identified tendency towards non-disclosure of CAM use to healthcare practitioners^35^ and the limited empirical evidence of efficacy for many CAM treatments, it is important to gain a fuller understanding of CAM use by older people with debilitating musculoskeletal pain. Further understanding of the combined usage of CAM alongside traditional treatments such as physiotherapy is also warranted. The present study uses data from a pan-European cross-sectional survey study to examine CAM use by older people with musculoskeletal disorders experiencing pain that hampers their daily living.

## Methods

### Study

This study is based on cross-sectional data from the 2014 round of the European Social Survey (ESS), a biennial pan-European survey, with data from 21 countries included. These countries included Austria, Belgium, Czech Republic, Denmark, Estonia, Finland, France, Germany, Hungry, Ireland, Israel, Lithuania, The Netherlands, Norway, Poland, Portugal, Slovenia, Spain, Sweden, Switzerland and the United Kingdom. Data were collected via face-to-face interviews with individuals aged 15 years and over living in private households. The average response level for all countries was 51.6%. Data from a total of 35,063 participants were collected. The 2014 round of ESS included a core module of substantive and socio-demographic items and a rotating module on the social determinants of health and health.

The ESS subscribes to the Declaration on Professional Ethics of the International Statistical Institute (ISI) (https://www.isi-web.org/about-isi/policies/professional-ethics/isi-declaration), to which the survey agencies that conduct the data collection adhere, in addition to any co-existing national obligations. No further ethical approval for the specific analyses presented here was needed.

### Sample

For this study, a sample of individuals from the 2014 European Social Survey dataset, aged 55 and older, who reported the presence of pain, as well as reporting that this pain hampers their daily activities, were investigated (n = 4950). The presence of pain was considered as any participants who reported a health problem with any one of the following, back or neck pain, muscular or joint pain in the hand or arm and muscular or joint pain in food or leg. While pain that hampers daily activities was considered as any participant who reported a health problem experienced in the last 12 months that hampered their daily activities in any way.

From a total of 35,063 individuals who took part in the ESS study, 13,016 (37%) were aged 55 or older; of which 8183 (63%) reported the presence of pain, with a further 4950 (38%) reporting that this pain hampered their daily activities in any way.

### Measures

#### Demographic information

A country was categorised into four groups as previously reported:^36^ ‘North’ (Denmark, Finland, Norway and Sweden), ‘West’ (Austria, Belgium, France, Germany, Ireland, The Netherlands, Switzerland and the United Kingdom), ‘Central/ East’ (Czech Republic, Estonia, Hungry, Lithuania, Poland and Slovenia) and ‘South’ (Israel, Portugal and Spain).

Other demographic information collected included: sex (male and female), and age (55–64, 65–74, 75–84 and 85+), employment (employed, retired and other) and education, classified using the International Standard Classification of Education (ISCED). Educational status was categorised as low secondary or less (ISCED I and II), upper secondary (ISCED IIIa, IIIb and IV) and tertiary (ISCED V), as per previous studies.^37^

#### Health problems

Participants were asked which of the health problems they experienced in the last 12 months: back or neck pain, muscular or joint pain in hand or arm or muscular or joint pain in foot or leg.

In terms of other physical health-related problems, individuals were asked which of the health problems they have had or experienced in the last 12 months, from a list of the following: heart or circulation problem, high blood pressure, breathing problems, stomach or digestion-related, skin condition-related, severe headaches, diabetes and cancer.

Co-occurring physical health problems was created by combining all physical health problems (heart or circulation problem, high blood pressure, breathing problems, stomach- or digestion-related, skin condition-related, severe headaches, diabetes and cancer). This was presented as a dichotomous variable (no, yes), where ‘yes’ represents individuals reporting pain and one or more co-occurring physical health problems.

Depression was assessed using an eight-item version of the Centre for Epidemiological Studies Depression Scale (CES-D scale).^38^ Individuals were asked how often they felt each of the following in the past week: felt depressed; felt everything was an effort; sleep was restless; was happy; felt lonely; enjoyed life; felt sad and could not get going. For this article, symptoms of depression were coded as those scoring a value of 10 or more.^36^

#### Healthcare utilisation

Individuals were asked if they had discussed their health with a general practitioner, or a medical specialist (excluding a dentist) in the last 12 months. Individuals were also asked which alternative health treatments they used in the last 12 months, from a list of the following: physiotherapy, acupuncture, acupressure, Chinese medicine, chiropractic, osteopathy, homoeopathy, herbal treatment, hypnotherapy, massage therapy, reflexology and spiritual healing.

The CAM treatments were categorised into the four therapy types as was previously operationalised by Kemppainen et al.^20^ The Traditional Asian Medical Systems (TAMS) category included traditional Chinese medicine, acupuncture and acupressure. The Alternative Medicinal Systems (AMSs) category included homoeopathy and herbal treatment. The Manual body-based therapies (MBBTs) category included massage therapy, chiropractic, osteopathy and reflexology; and the Mind-Body Therapies (MBTs) category included hypnotherapy and spiritual healing.

Each group was dichotomised to represent whether the treatments were used during the last 12 months or not. Physiotherapy was not included in one of the four therapy types as it is not typically considered a complementary or alternative therapy.

### Statistical analysis

Categorical data were described using counts and percentages. Continuous data that approximated a normal distribution were described using means and standard deviations. Pearson’s Chi Square test was used to test differences between categorical variables. Cramer’s V effect size was reported, with V = 0.1, 0.3 and 0.5 for a small, medium and large effects, respectively. Both post-stratification and population weights have been applied for analysis pooling data across countries to give all countries a weight proportional to population size.^39^ A 5% level of significance was used for all statistical tests. All statistical analysis was undertaken using SPSS Version 24.

## Results

### Descriptive statistics

Table 1 presents demographic information on the sample investigated (n = 4950). Of those who reported pain that hampers their daily lives, 1930 (39.0%) were male and 3018 (61.0%) were female. The majority (40.4%) of the older adults were in the 55- to 64-age band. Older adults aged 85+ represented 6.7% of the sample (n = 333). The median age was 67 years (IQR = 14). The majority of the sample (62.2%) were retired. Of those who reported pain that hampers their daily lives, most (63.5%) were from the West of Europe. The highest proportion of older adults reported low secondary or less education (47.2%).

**Table 1.** Demographic information of participants (n = 4950).

Sex	Male	1930 (39.0)
	Female	3018 (61.0)
Age	55–64	1998 (40.4)
	65–74	1584 (32.0)
	75–84	1035 (20.9)
	85+	333 (6.7)
Employment	Employed	1017 (20.6)
	Retired	3073 (62.2)
	Other	850 (17.2)
Education	Lower secondary or less	2334 (47.2)
	Upper secondary	2024 (40.9)
	Tertiary	542 (10.9)
Country	North	292 (5.9)
	West	3143 (63.5)
	Central/East	926 (18.7)
	South	589 (11.9)

### Differences in pain and co-occurrence of other health-related problems, by age

Table 2 presents the occurrence of specific musculoskeletal pain sites for the whole sample and across age bands. In general, there was a high prevalence for each of the three musculoskeletal pain sites (back/neck, hand/arm and foot/leg) across the sample; 68.9% reported back or neck pain, 55.9% reported muscular or joint pain in the hand or arm and 60.6% reported muscular or joint pain in the foot or leg. There were no differences across age groups in terms of hand or arm pain. However, younger old adults (aged 55–74) were more likely to report back or neck pain. While the older groups (aged 75+) were more likely to report foot or leg pain.

**Table 2.** Differences in reported health problems by age group.

		Full sample (n = 4950)	Age				*p* value (effect size)
			55–64 (n = 1998)	65–74 (n = 1584)	75–84 (n = 1035)	85+ (n = 333)	
Back/neck pain	Yes	3410 (68.9)	1475 (73.8)	1080 (68.2)	652 (63.0)	203 (61.1)	<0.001 (0.10)
Muscular or joint pain in the hand or arm	Yes	2769 (55.9)	1082 (54.2)	884 (55.8)	612 (59.1)	191 (57.4)	0.07 (0.04)
Muscular or joint pain in the foot or leg	Yes	3001 (60.6)	1041 (52.1)	1010 (63.8)	723 (69.9)	227 (68.2)	<0.001 (0.15)
Co-occurring physical health problems	Yes	3593 (74.6)	1309 (67.2)	1178 (77.1)	837 (82.5)	270 (82.1)	<0.001 (0.15)
Physical health problems							
Heart or circulation problem	Yes	1333 (26.9)	332 (16.6)	443 (28.0)	412 (39.8)	146 (44.0)	<0.001 (0.22)
High blood pressure	Yes	2011 (40.6)	612 (30.6)	714 (45.1)	531 (51.3)	154 (46.2)	<0.001 (0.17)
Breathing problems	Yes	786 (15.9)	286 (14.3)	222 (14.0)	198 (19.1)	80 (24.0)	<0.001 (0.08)
Stomach- or digestion-related	Yes	1053 (21.3)	424 (21.2)	358 (22.6)	227 (21.9)	44 (13.2)	0.002 (0.06)
Skin condition	Yes	564 (11.4)	225 (11.3)	182 (11.5)	124 (12.0)	33 (9.9)	0.77 (0.02)
Severe headache	Yes	720 (14.5)	340 (17.0)	207 (13.1)	133 (12.90)	40 (12.0)	0.001 (0.060)
Diabetes	Yes	718 (14.5)	184 (9.2)	279 (17.6)	204 (19.7)	52 (15.6)	<0.001 (0.13)
Cancer (currently)	Yes	375 (7.8)	94 (4.8)	142 (9.3)	99 (9.8)	29 (11.9)	<0.001 (0.10)
Depression	Yes	959 (19.8)	359 (18.2)	270 (17.4)	251 (25.3)	79 (24.5)	<0.001 (0.08)

Count (%) presented. Cramer’s V effect size.

Health problems, split by age, are also presented in Table 2; 74.6% of people who reported pain that hampers daily life also reported at least one other physical health-related problem. Statistically significant differences were observed between age groups, across all co-occurring physical health problems, except for skin conditions, and symptoms of depression.

In the older age categories (ages 75+), certain health concerns tended to be more prevalent such as heart or circulation problems (83.8%), high blood pressure (97.5%), breathing problems (43.1%), diabetes (35.3%), cancer (21.7%) and symptoms of depression (49.8%). In contrast, in the younger adults (aged 55–74), stomach- or digestion-related health problems (43.8%) and severe headaches (30.1%) were more prevalent.

### Difference in utilisation of healthcare by age

Table 3 presents healthcare utilisation, by age. The older adults, aged 85+, tend to utilise healthcare, such as general practitioners and medical specialists, more than the younger old, aged 55–65. For example, 86.5% of older adults aged 55–64, visited a GP in the previous year, compared to 95.8% of 85+ year olds.

**Table 3.** Differences in reported healthcare utilisation, by age group.

	Full sample (n = 4950)	Age				*p* value (effect size)
		55–64 (n = 1998)	65–74 (n = 1584)	75–84 (n = 1035)	85+ (n = 333)	
Reported healthcare utilization						
General practitioner	4491 (90.7)	1729 (86.5)	1447 (91.4)	995 (96.1)	319 (95.8)	<0.001 (0.13)
Medical specialist	3237 (65.4)	1233 (61.7)	1041 (65.7)	743 (71.8)	220 (66.1)	<0.001 (0.08)
Physiotherapy	1399 (28.3)	623 (31.2)	468 (29.6)	241 (23.3)	67 (20.1)	<0.001 (0.08)
Unable to get medical consultation or treatment (last 12 months)	686 (13.9)	371 (18.6)	185 (11.7)	104 (10.1)	26 (7.9)	<0.001 (0.12)
Reasons for unavailable consultation or treatment						
Waiting list too long	300 (6.1)	151 (7.6)	85 (5.4)	54 (5.2)	11 (3.3)	0.002 (0.06)
No appointments available	278 (5.6)	150 (7.5)	68 (4.3)	48 (4.6)	13 (3.9)	<0.001 (0.07)
Could not pay	85 (1.7)	43 (2.2)	28 (1.8)	11 (1.1)	3 (0.9)	0.10 (0.04)
Not available near home	85 (1.7)	43 (2.2)	22 (1.4)	18 (1.7)	3 (0.9)	0.21 (0.03)
Other*	149 (3.0)	89 (4.5)	40 (2.5)	16 (1.5)	4 (1.2)	<0.001 (0.08)

Count (%) presented. Cramer’s V effect size.

* Other included could not take time off work, other commitments and other reason.

In the whole sample, 28.3% reported physiotherapy use, with the younger old, aged 55–65, using this treatment more than the older adults aged 85+ (31.2% compared to 20.1%, respectively).

Of the older adults with pain that hampered daily activities, 13.9% report being unable to access medical consultation or treatments in the last 12 months. The main reasons were that the waiting lists were too long (6.1% yes response) and there is no appointments available (5.6% yes response). The younger categories were more likely to report waiting lists being too long and no appointments being available, than the older adults. No differences between age groups in terms of location availability or not being able to pay were noted.

### Socio-demographic and health differences in use of CAM treatments

Table 4 presents the use of CAM treatments in a sample of older adults reporting pain that hampers their daily activities. Of the whole sample, 1657 (33.5%) reported use of at least one CAM treatment. MBBTs were the most prevalent with 17.9% of older adults reporting massage therapy and 7.0% reporting osteopathy. AMSs were also popular, with 6.5% reporting homoeopathy use and 5.3% reporting herbal treatments.

**Table 4.** Differences in CAM treatment, by reported physiotherapy use.

CAM treatments	Full sample (n = 4950)	Physiotherapy		*p* value (effect size)
		No (n = 3550, 71.7%)	Yes (n = 1399, 28.3%)	
Traditional Asian medical systems				
Acupuncture	239 (4.8)	126 (3.5)	113 (8.1)	<0.001 (0.10)
Acupressure	41 (0.8)	21 (0.6)	20 (1.4)	0.003 (0.04)
Chinese medicine	50 (1.0)	19 (0.5)	31 (2.2)	<0.001 (0.08)
Alternative medicinal systems				
Homoeopathy	321 (6.5)	184 (5.2)	137 (9.8)	<0.001 (0.08)
Herbal treatment	262 (5.3)	171 (4.8)	91 (6.5)	0.02 (0.03)
Manual body-based therapies				
Massage therapy	886 (17.9)	444 (12.5)	442 (31.6)	<0.001 (0.22)
Chiropractic	156 (3.2)	83 (2.3)	73 (5.2)	<0.001 (0.07)
Osteopathy	345 (7.0)	181 (5.1)	163 (11.7)	<0.001 (0.12)
Reflexology	111 (2.3)	59 (1.7)	52 (3.7)	<0.001 (0.06)
Mind-body therapies				
Hypnotherapy	15 (0.3)	8 (0.2)	7 (0.5)	0.11 (0.02)
Spiritual healing	73 (1.5)	44 (1.2)	30 (2.1)	0.02 (0.03)

CAM: complementary and alternative medicine.

Count (%) presented. Cramer’s V effect size.

Physiotherapy use was reported by 1399 (28.2%) older adults with pain that hampers their daily lives, while 1657 (33.5%) reported use of at least one CAM treatment. Results suggest that those using physiotherapy are more likely to also use CAM treatments (Table 4). For example, 8.1% of those reporting physiotherapy also use acupuncture, compared to 3.5% who do not report physiotherapy use but use acupuncture.

Table 5 presents the socio-demographic differences in terms of use of CAM treatments, in a sample of older adults reporting pain that hampers daily life. Greater uptake of CAM treatments is observed among younger women, with higher levels of education, who are not retired and are from West Europe. There is an upward trend between CAM use and education, with those reporting higher levels of education more likely to use CAM treatments.

**Table 5.** Differences in CAM use by socio-demographics and health issues.

		TAMS	*p* value	AMS	*p* value	MBBT	*p* value	MBT	*p* value
		(n = 305, 6.2%)	(effect size)	(n = 507, 10.2%)	(effect size)	(n = 1257, 25.4%)	(effect size)	(n = 85, 1.7%)	(effect size)
Sex	Male	94 (4.9)	0.002 (0.04)	128 (6.6)	<0.001 (0.10)	416 (21.6)	<0.001 (0.07)	35 (1.8)	0.68 (0.01)
	Female	211 (7.0)		379 (12.6)		842 (27.9)		50 (1.7)	
Age	55–64	136 (6.8)	0.003 (0.05)	225 (11.3)	0.001 (0.06)	585 (29.3)	<0.001 (0.08)	52 (2.6)	<0.001 (0.07)
	65–74	112 (7.1)		179 (11.3)		393 (24.8)		25 (1.6)	
	75–84	47 (4.5)		85 (8.2)		215 (20.8)		4 (0.4)	
	85+	10 (3.0)		19 (5.7)		65 (19.5)		4 (1.2)	
Education	Lower secondary or less	98 (4.2)	<0.001 (0.08)	164 (7.0)	<0.001 (0.11)	404 (17.3)	<0.001 (0.19)	24 (1.0)	<0.001 (0.06)
	Upper secondary	154 (7.6)		255 (12.6)		643 (31.8)		43 (2.1)	
	Tertiary	48 (8.9)		87 (16.1)		205 (37.8)		18 (3.3)	
Employment	Employed	78 (7.7)	0.005 (0.05)	129 (12.7)	0.006 (0.05)	325 (32.0)	<0.001 (0.08)	33 (3.2)	<0.001 (0.07)
	Retired	163 (5.3)		284 (9.2)		717 (23.3)		32 (1.0)	
	Other	64 (7.5)		91 (10.7)		211 (24.8)		19 (2.2)	
Country	North	26 (8.9)	< 0.001 (0.12)	8 (2.7)	<0.001 (0.09)	80 (27.3)	<0.001 (0.17)	6 (2.0)	0.55 (0.02)
	West	248 (7.9)		377 (12.0)		955 (30.4)		59 (1.9)	
	Central/East	17 (1.8)		87(9.4)		157 (17.0)		12 (1.3)	
	South	13 (2.2)		35 (5.9)		66 (11.2)		8 (1.4)	
Co-occurring physical health problem	No	54 (4.4)	0.002 (0.04)	121 (9.9)	0.76 (0.00)	311 (25.4)	0.91 (0.00)	11 (0.9)	0.01 (0.04)
	Yes	246 (6.8)		366 (10.2)		907 (25.2)		70 (1.9)	
Depression	No	241 (6.2)	0.75 (0.01)	388 (10.0)	0.45 (0.01)	1034 (26.7)	0.002 (0.05)	62 (1.6)	0.14 (0.02)
	Yes	57 (5.9)		104 (10.8)		208 (21.7)		22 (2.3)	

TAMS: traditional Asian medical systems; MBBTs: manual body-based therapies; MBTs: mind-body therapies.

Count (%) presented. Cramer’s V effect size.

Those suffering from multiple physical health problems were more likely to use all CAM treatments, except MBBT, where there was no difference between individuals suffering from multiple health-related problems and those only reporting pain that hampers daily life. The only significant difference between those who suffer from depressive symptoms and those who do not is in the use of MBBT, with those not depressed more likely to use MBBT (26.7% compared to 21.7%).

## Discussion

Of the older adult participants who reported pain in the ESS, 4950 (60%) reported that this pain hampers their daily activities. Most of this sample further reported at least one other physical health-related problem (74.6%). MBBTs such as massage therapy and osteopathy were identified as the most commonly used CAM by older people with hampering pain followed by AMS (including homoeopathy and herbal treatments). CAM use was associated with physiotherapy use, female gender, younger age, higher levels of education, being in employment and living in West Europe. Those reporting multiple physical health problems were more likely to use the TAMS and MBBT, compared to individuals only reporting pain that hampers their daily lives.

Within this study, 63% of older adults reported pain, while 38% of older adults reported pain that hampers their daily lives. These findings are reflective of findings from The Survey of Health, Ageing and Retirement which reported that at wave 4, 57.4% of older adults suffered from pain, across 13 European countries.^40^ Elsewhere, however, other European studies have reported lower rates of pain among older people. For example, The Irish Longitudinal Study on Ageing (TILDA) reported that 36% of older Irish adults were ‘often troubled with pain’,^41^ and a study drawing on waves 2–8 of The English Study on Ageing (ELSA) reported that 35.7% older English adults were ‘often troubled with pain’.^42^ In general, across these three European population studies, women with lower rates of education were more likely to report pain.^40,41^ More recently, the whole ESS sample was investigated,^43^ and while the overall prevalence of pain was lower once the younger population were included, again, similar to this study, women, people reporting lower education levels, and people from West and North Europe, were more likely to report pain. Interestingly, Todd et al.^43^ also reflect the lower pain rates in Ireland and England in comparison to other West European countries, which is consistent with the results published by TILDA and ELSA.

ESS data have shown 25.9% of the general European population across all age groups to report CAM use.^20^ In our study, we found that this figure increases to 33.5% among older adults experiencing hampering musculoskeletal pain. Our findings reflect other studies of CAM use among older people in the United States, which have reported high rates of CAM use of between 23% and 62.9%.^13,44^ In both the general population^20,21^ and older adult groups,^45^ women and those with higher education are most likely to use CAM, in line with our results. Other studies in various regions have also found women^46,47^ and those with higher education^48^ to be more likely to use CAM.

Most respondents in this study reporting musculoskeletal pain also reported at least one other physical health-related problem (74.6%). Health concerns such as heart or circulation problems, high blood pressure, breathing problems, diabetes, cancer and symptoms of depression tended to be more prevalent in the older age categories. Older people with co-existing other health conditions in this study were more likely to use all CAM categories of treatment. Using data from the 2012 National Health Interview Survey, Alwhaibi et al.^49^ found that people with co-existing physical and mental health conditions are more likely to use CAM than people with co-existing physical conditions.

In this study, older people from West European countries reported the highest rates of CAM use compared to participants from North, Central/East or South regions. Patterns of CAM use have been found to differ among racial/ethnic groups^50^ and national differences in specific CAM therapy preference have been repeatedly reported.^21,46,51^ CAM is frequently used in Germany where CAM delivered by non-physicians has been legally regulated since 1939.^52^ A systematic review of 16 surveys found CAM use rates in the previous year in Germany ranging from 40% and 62% of the general adult population.^53^ International variability in the funding of CAM interventions possibly influences geographical variation in CAM use. In Switzerland, for example, five CAM methods are covered by the mandatory basic health insurance when performed by a certified physician (traditional Chinese medicine/acupuncture, homoeopathy, anthroposophic medicine, neural therapy and herbal medicine).^54^

### Strengths and limitations

Strengths of this study are that it utilises data from a large pan-European study, of 21 countries, providing useful insights into the use of CAM in older adults who report pain that hampers their daily lives. However, the findings of this study are still somewhat limited, due to the self-reported nature of the ESS data, as well as the lack of data collected. For example, musculoskeletal pain was self-reported via a single item, while no information was reported on frequency of CAM use and specific reasons for CAM use. Given the very high rates of other conditions reported alongside musculoskeletal pain, it is impossible to know if CAM was used by respondents for pain or another condition or for other reasons, for example, relaxation, mood or well-being. No data was available on spending associated with CAM use, reimbursement of CAM or disclosure of CAM to healthcare providers. Comparability of findings from various CAM studies is hampered by the varied definitions and diverse categories used within studies. Wide variation exists in what constitutes a CAM therapy with variations of prayer, supplements, rubs, lotions, relaxation exercises, copper bracelets, thermal therapies and meditation included in definitions of CAM.^55,56^ The ESS categorised CAM into four main categories which did not include biologically based therapies (non-vitamin and non-mineral supplements) – another common category of CAM for older adults across other studies.^57,58^ Consistent with other papers using ESS data, we grouped countries into four regions (North, West, East and South) for analysis; however, the association between pain and CAM use may differ between countries within each region. A further limitation is that ESS does not include participants living in institutional settings.

### Implications for future research and practice

Despite high rates of CAM use, there has been a lack of attention to CAM or how to support patient disclosure of CAM in clinical practice guidelines or editorials on pain management^59^ or in guidelines on the assessment of pain in older people.^60^ Clinical implications arising from this study include the need for clinicians to support patients to disclose CAM use and comprehensively and routinely assess for CAM use in older adults with musculoskeletal pain. Future research could explore the specific indications for CAM use, and explore potential associations with reimbursement patterns nationally. Research is also needed to identify how CAM use is combined with, or replaces, more traditional pain management strategies (e.g. physiotherapy and pharmacology) among older adults.

## Conclusion

A third of older Europeans, who report pain that hampers their daily lives, report CAM use in the previous 12 months, with MBBT the most popular. This study identified certain subgroups with higher rates of CAM use; specifically, physiotherapy use, female gender, younger age, higher levels of education, being in employment and living in West Europe. Clinicians should comprehensively and routinely assess CAM use among older adults with musculoskeletal pain while being cognisant of the importance of supporting patient disclosure of CAM use.
